# The histocompatibility research of hair follicle stem cells with bladder acellular matrix

**DOI:** 10.1097/MD.0000000000004979

**Published:** 2016-11-11

**Authors:** Jia Li, Wenguang Wang, Jiuzhi Li, Mulati Rexiati, Henqing An, Feng Wang, Yujie Wang

**Affiliations:** a Urinary Center and Pediatric Center, The First Affiliated Hospital of Xinjiang Medical University; b Department of Urology, Renmin Hospital of Xinjiang Uygur Autonomous Region; c Urinary Center, The First Affiliated Hospital of Xinjiang Medical University, Urumqi City, Xinjiang, China.

**Keywords:** bladder acellular matrix, hair follicle stem cells, histocompatibility

## Abstract

**Background:**

Hair follicle stem cells (HFSCs) were reported to have multidirectional differentiation ability and could be differentiated into melanocytes, keratin cells, smooth muscle cells, and neurons. However, the functionality of HFSCs in bladder tissue regeneration is unknown.

**Methods:**

This study was conducted to build HFSCs vs bladder acellular matrix (BAM) complexes (HFSCs–BAM complexes) in vitro and evaluated whether HFSCs have well biocompatibility with BAM. HFSCs were separated from SD rats. BAM scaffold was prepared from the submucosa of rabbit bladder tissue. Afterwards, HFSCs were inoculated on BAM.

**Results:**

HFSCs–BAM complexes grew rapidly through inverted microscope observation. Cell growth curve showed the proliferation was in stagnate phase at 7th and 8th day. Cytotoxicity assay showed the toxicity grading of BAM was 0 or 1. Scanning electron microscopy, HE staining, and masson staining showed that cells have germinated on the surface of scaffold.

**Conclusion:**

The results provide evidence that HFSCs–BAM complexes have well biocompatibility and accumulate important experimental basis for clinical applying of tissue engineering bladder.

## 1 Introduction

The structure and function defects of bladder may cause by trauma, congenital malformation, tumor, and other diseases, which make the patients suffer from renal insufficiency and renal failure. Traditional bladder tissue repair materials contain an autologous nonurinary system, such as gastrointestinal tract tissue, and synthetic polymers or nature derivative materials, such as poly hydroxy acetic acid and collagen.^[1]^ Recent tissue engineering researches of bladder repair and reconstruction have got a remarkable progress.^[2]^ The establishment of tissue engineering bladder is mainly based on 3 major technologies that are the appropriate seed cells, the appropriate scaffold materials, the implantation of seed cells with scaffold materials and induced into target tissue or organ. The third section is the focus of present research and still in the stage of exploration. Currently, one of the research directions of tissue engineering is that inducing pluripotent stem cells or adult stem cells into the epithelial cells and smooth muscle cells, which is the research priorities of establishment of tissue engineering bladder.

In recent years, researchers have found that HFSCs, existing in the outer root sheath of hair follicle, have been shown to be an excellent resource of regenerative stem cells with multidirectional differentiation ability.^[3]^ In vitro, HFSCs could be differentiated into melanocytes, keratin cells, smooth muscle cells, and neurons. Besides, it could express strong immune suppression effectiveness and decrease the immune activity of histocompatibility protein.^[4,5]^ In the present study, we evaluated whether HFSCs have favourable biocompatibility with BAM.

## 2 Methods

### 2.1 Acquisition of HFSCs from SD rats

Clean SD young rats (birth 7–9 days) provided by the Xinjiang medical university animal center were killed by cervical dislocation. This research has been approved by Xinjiang medical university animal ethics committee. After the tentacles of rats were cut off with ophthalmic scissors, the skin was disinfected by 5% iodine volts solution and 75% alcohol solution 3 times separately. The skin and subcutaneous tissue was sheared with sterile ophthalmic scissors and put into PBS buffer (containing 5% amphotericin B and 5% streptomycin) in sterile glass bottle. The tissue was rinsed with 75% alcohol solution for 30 seconds 3 times in clean bench and soaked with PBS buffer (containing 5% amphotericin B and 5% streptomycin) for 2 to 3 minutes 3 times. It was very important to ensure aseptic in the whole process. While the tissue was cut into 0.5 × 0.5 cm^2^ size, hair follicle tissue was extracted completely with ophthalmic scissors under microscope. The hair follicle tissue was put in 10 mL centrifugal tube which was added with 5 mL 5 mg/mL Dispase II solution. The closed centrifugal tube that digested on the shaking table for 2 hours 100 r/min at 37°C was centrifuged at 2000 rpm. The supernatant was discarded after centrifugation. The precipitate was rinsed with pure PBS solution 3 times to wipe off neutral protease and digested with 0.125% trypsin + 0.01% EDTA solution 3 mL for 30 minutes on shaking table 100 r/min at 37°C. As soon as the hair follicle tissue was broken down into floc, 10% fetal bovine serum was added to terminate the digestion. The cells and suspension were filtered by 200-mesh sieve. The filtrate was transferred into 10 mL centrifugal tube and centrifuged 1500 rpm for 10 minutes. The cells were seeded in the culture bottle that packed with IV collagen at the concentration of 5 × 10^4^/mL. The bottle was cultured in incubator at 37°C 5% CO_2_.

### 2.2 HFSCs amplification

HFSCs were grouped by differential adhesion. The culture bottle was shaked in cross-shape equably and placed in the CO_2_ incubator for 30 minutes. Then, the bottle was added K-FSM+10%FBS culture solution after supernatant was removed. The culture solution was replaced every 2 to 3 days based on cell growth. When cell growth rate was nearly 80%, the cell proliferation was in logarithmic phase and need to be amplificated. After culture solution was removed, the cells were rinsed 3 times with PBS and added with 1 mL 0.125% trypsin-0.0l% EDTA digestive solution. The sealed culture bottle was static culture in incubator for 15 minutes at 37°C. Then, the cells were added 10 mL culture solution (containing 10% fetal bovine serum) to terminate the digestion. Cell suspension was moved into 15 mL centrifuge tube and centrifuged 1500 rpm for 5 minutes. After the supernate was discarded, the precipitate was added 1 mL culture solution to blend. The cells were seeded in the 25 mL culture bottle packaged with IV collagen at concentration of 5 × 10^4^/mL. The culture bottle was marked with generation times and cultured in incubator.

### 2.3 Extracting submucosa from rabbit bladder

The bladder was got from new Zealand white rabbits which were executed with air embolism and the surrounding fascia and adipose tissue was cleaned up. Afterwards, the bladder was repeatedly rinsed 3 times with D-Hanks buffer (containing 10% penicillin-streptomycin) and preserved in the D-Hanks buffer at 4°C. The incision on the bladder was longitudinal and the mucosal surface was upward. When the mucosa layer of bladder was scraped carefully, the submucosa and mucosa muscular layers were separated carefully by microscopic scissor and microscopic tweezers. The operator must maintain the integrity of submucosa in the procedure. The segregated submucosa was cut into the size of 1 cm^2^ and rinsed 3 times with pure PBS solution.

### 2.4 Fabrication of BAM

The submucosa that was acquired in the previous assay was put in PBS buffer (containing 0.1% sodium azide) and oscillated overnight at a speed of 250 rpm/minutes. After rinsed 3 times with PBS buffer, the submucosa was put in PBS buffer (containing 100 mL 0.4% trypsin and 0.5 mmol/L EDTA) and stirred at room temperature 5 to 6 hours at a speed of 250 rpm/min in order to break cellular structure. After rinsed 3 times with PBS buffer, the submucosa was put in 100 mL 1 mol/L sodium chloride solution (containing 4000kU DNase-1) and stirred at 37°C 6 to 8 hours at a speed of 250 rpm/minutes in order to eliminate nucleic acid composition completely. BAM was put in 100 mL PBS buffer (containing 0.1%Sodium azide and 4% sodium deoxycholate) and stirred at 37°C 6 to 8 hours at a speed of 250 rpm/minutes in order to eliminate cell component. BAM was placed in the vacuum freeze drier 6 h in 24-well palate after rinsed with PBS buffer (containing 5% penicillin-streptomycin). The freeze-dried BAM was dealed with radiation sterilization by Cobalt 60 at 25 kgray dose. It is important to note that all the operation must be kept in the sterile operation.

### 2.5 Fabrication of HFSCs–BAM complexes

Prepared rabbit BAM was rinsed with sterile PBS buffer 3 times, each time for 5 minutes. The scaffold was put in 24-well plate added with culture solution and placed in incubator for 24 hours at 37°C and 5% CO_2_. 0.5 mL of cell suspension, 1 × 106/mL of concentration, was added into the 24-well plate. Then, the plate was placed in incubator once again for 30 minutes. 0.5 mL of culture solution was added again. 30 minutes later, 1 mL of culture solution was added and the plate was placed in incubator once more.

## 3 Results

### 3.1 Culture of HFSCs

**Figure 1:**
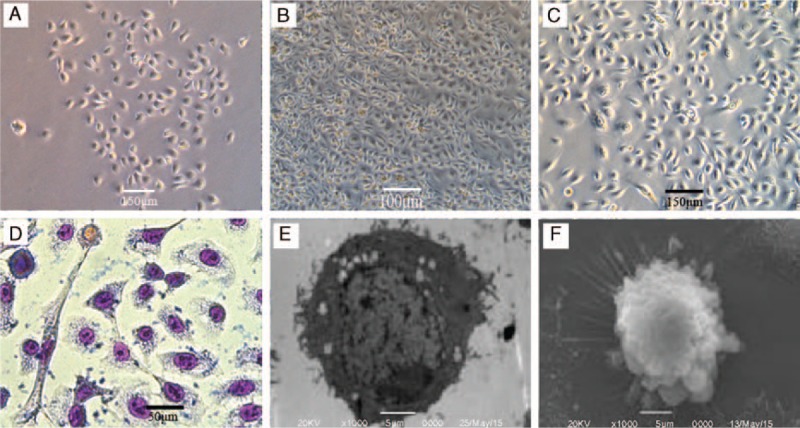
Cellular morphology under the inverted phase contrast microscope. (A) 48 hours, polygonous adherent cell and spindle fibroblasts diffused distribution (50×). (B) 6 days, cells covered about 60% of the bottom of culture bottle (50×). (C) The 3rd generation of HFSCs (100×). (D) Giemsa staining of the 3rd generation of HFSCs (400×). (E) Cells under the transmission electron microscope (1000×). (F) Cells under the scanning electron microscope (1000×). HFSCs = hair follicle stem cells.

After hair follicle tissue digestion, the cells of suspension were inoculated into culture bottle packaged with IV colleagen. HFSCs were one kind of stem cells with active proliferation ability. It was expected to grow well in appropriate culture medium. The bottle was put in incubator. For 48 hours, the polygonous adherent cell was diffused distribution with spindle fibroblasts and other types of cells mingled (Fig. 1A). For 6 days, cells covered ∼60% of the bottom of culture bottle (Fig. 1B). Using the method of differential adhesion, the spindle fibroblasts were eliminated gradually. At the 3rd generation, cellular morphology trend to accord, presently owl-eye cell (Fig. 1C). Giemsa staining in the 5th day, cytoplasmic was purple, and nucleus was pink under microscope. Cell colony form a very regular “pavement” when viewed from the surface (Fig. 1D). Transmission electron microscope showed that cells volume was small, caryoplasm ratio was large, organelles was immature, nucleolus was obvious. These characteristics demonstrated that stem cells were in initial condition (Fig. 1E). The scanning electron microscope showed that there was villus attaching on the cells (Fig. 1F).

### 3.2 Microscopy observation of BAM

**Figure 2:**
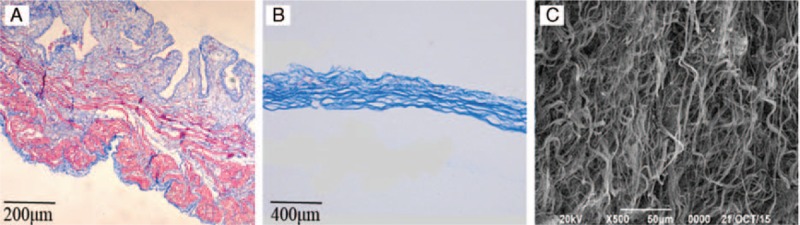
Microscope observation of bladde. (A) The Masson staining of rabbit bladde (100×). (B) The Masson staining of BAM (100×). (C) Scanning electron microscopy demonstrated that there were no cells on the surface of BAM (500×). BAM = bladder acellular matrix.

The living bladder was suitable for regeneration scaffold with high elasticity. Masson staining showed that the anatomic structure of original rabbit bladder was clear. The muscular fiber layer was red-staining and different stratum arranged regularly (Fig. 2A). However, the processed BAM showed the homogeneous state blue layer which was collagen fibers without cells (Fig. 2B). Under the scanning electron microscope, the prepared BAM was fibered network. Irregular space disperses in the reticular structure. There were no residual cells on the surface of BAM (Fig. 2C).

### 3.3 Cytotoxicity assay of BAM

The absorbance (symbolic name “A”) value was measured with the CCK-8 method. Different concentrations of BAM leaching solution were added into cell culture medium at 1st, 2nd, 3rd, 4th, 5th day. The calculation of relative growth rate (RGR) was >80% and cell toxicity grade was 0 or 1 (Table 1).

**Table 1:**
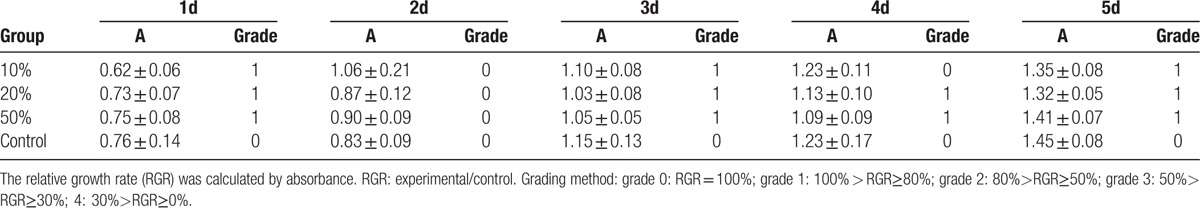
The cytotoxicity of BAM was measured with CCK-8. “A” represent absorbance.

### 3.4 HFSCs–BAM complexes compatibility assay in vitro

**Figure 3:**
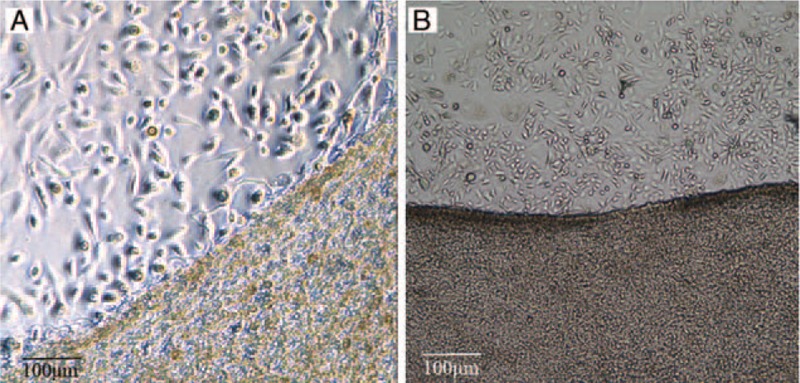
Inverted microscope observation of HFSCs-BAM complexes. (A) HFSCs-BAM complexes culture for 2 hours in vitro (100×). (B) HFSCs-BAM complexes culture for 5 days in vitro (50×). BAM = bladder acellular matrix, HFSCs = hair follicle stem cells.

BAM acted as the scaffold for the HFSCs. The morphology and consistency of HFSCs represented the growth situation. 2 hours later, a large number of cells attached to scaffold surface and bottom of petri dish under inverted microscope. Cell morphology was oval or round (Fig. 3A). After 48 hours, the cells adhered to scaffold was spindle, arraying consistently. The bottom cells grew well, presenting regular “pavement” when viewed from inverted microscope. 5 days later, the bottom cells merging exceeding 80%. The cells on the scaffold surface increased obviously (Fig. 3B).

### 3.5 Cell growth curve of HFSCs–BAM complexes

**Figure 4:**
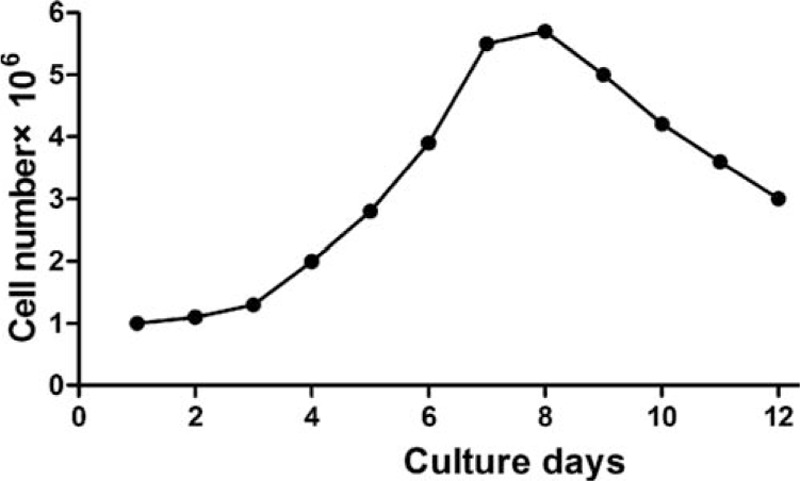
Cell growth curve of HFSCs-BAM complexes. Cells, which were digested with 0.125% trypsin+0.0l % EDTA and centrifuged, were counted under microscope. BAM = bladder acellular matrix, HFSCs = hair follicle stem cells.

Cell number was counted under microscope. The quantity of HFSCs reflected the growth status. In the HFSCs–BAM complexes culture, the growth curve was consistent. Cell growth was in stagnate phase at 7 to 8 days (Fig. 4). These results suggested that HFSCs’ growth was in good condition on the surface of BAM scaffold. HFSCs and BAM scaffold had good biocompatibility.

### 3.6 Scanning electron microscopy and histological examination of HFSCs–BAM complexes

**Figure 5:**
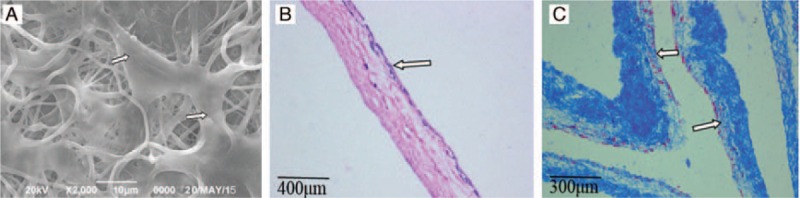
HFSCs-BAM complexes in the 7th day in vitro. (A) scanning electron microscopy (2000×). (B) HE staining (100×). (C) Masson staining (100 × ). Arrows showed the cells on the surface of scaffold. BAM = bladder acellular matrix, HFSCs = hair follicle stem cells.

With continuous culture, HFSCs were in good combination with BAM. In the 7th day in vitro, HFSCs–BAM complexes were fixed by paraformaldehyde. After HE staining and Masson staining, there were 1 to 3 layers of cells on the surface of the scaffold under microscope (Fig. 5).

## 4 Discussion

### 4.1 Biological characteristics of HFSCs

Stem cells have the ability of replicate and multidirectional differentiation potential. There are three characteristics: self-renewal capacity, differentiation ability, and asexual reproduction ability.^[6]^ HFSCs is a kind of adult stem cells in hair follicles that widely distributes in body surface with sufficient source. Hair follicle is the subsidiary organs of skin, which has unique organization and structure, having periodic regeneration ability in the whole mammals life process. According to study of recent years, HFSCs is the foundation to maintain the renewal of hair follicle. HFSCs has 2 most significant features: self-renewal ability and slow periodicity. In 1981, Cotsarelis G. confirmed that HFSCs act as label retaining cells in the body, which belongs to slow periodicity.^[7]^ Under the proper condition, HFSCs still has very strong proliferation ability >20 generation.^[8]^ Scholars have found that HFSCs has the ability of migration.^[9]^ There are several methods for separation and purification of HFSCs in vitro, such as enzyme digestion, tissue block, flow cytometry separation, differential attachment, microseparation, and so on.^[10–12]^ Our study found a effective method to obtain high purity and good proliferation activity HFSCs, which were microseparation + two-step enzyme digestion + differential attachment. The cellular morphology of HFSCs was “owl-eye” at the 3rd generations (Fig. 1C). Cell colony formed a very regular “pavement” with giemsa staining in the 5th day (Fig. 1D). Transmission electron microscope demonstrated that HFSCs were in initial condition (Fig. 1E). These cells have differentiation potential to differentiate into adult cell. Currently, one of the research direction of tissue engineering is to induce stem cells to re-establish bladder.^[13,14]^

### 4.2 The selection of tissue engineering bladder scaffold

Appropriate scaffold is the foundation to construct tissue engineering bladder, which affect morphology and function of bladder and play the decisive role. At present, there is no generally accepted materials for constructing clinical tissue engineering bladder.^[15]^ Tissue engineering scaffold materials contains synthetic materials and decellularized tissue matrix materials. Decellularized tissue matrix materials have been increasingly applied to the urinary bladder reconstruction research.^[16]^ Currently, the major applied materials contain decellularized amnion, bladder decellularized matrix, and small intestine submucosa.^[17]^

Our study selects the BAM to serve as tissue engineering scaffold to reconstruct bladder in vitro. BAM shows more advantages than other materials.^[18]^ The absorption rate and degradation rate of BAM were the same with normal bladder tissues.^[19]^ The chemical properties of BAM surface is more suitable for cells adsorption and growth, which provide favourable regeneration scaffold for bladder mucosa, smooth muscle, blood vessels, and nerve.^[20]^ BAM has good biocompatibility with graft host and seed cells and seldomly produce rejection reaction.^[21]^ In 2006, Atala applied autologous urinary tract and smooth muscle cells to repair the defect of human bladder, which was a landmark in the history of the bladder reconstruction.^[22]^ Researches have pointed out that BAM contains a variety of tissue healing and regeneration growth factors, mainly including transforming growth factor beta (TGF-beta), fibroblast growth factor (FGF) and vascular endothelial growth factor (VEGF) and so on, that promote the induction of seed cell differentiatio.^[23]^ In our study, the fabrication method of BAM is combined with the existing research. In masson staining, the BAM showed homogeneous state blue layer that was collagen fibers without cells (Fig. 2B). Under scanning electron microscope, the prepared BAM is reticular structure with irregular space dispersing (Fig. 2C). Above results show that the acellular bladder submucosa, as a kind of natural biological material, is a kind of ideal scaffold materials for tissue engineering.

### 4.3 The compatibility of HFSCs–BAM complexes

The biocompatibility between biological materials and cells is one of the most important elements to evaluate the quality of biological materials. Good biocompatibility is the essential condition for tissue engineering materials. Our study has observed the biocompatibility of HFSCs and BAM scaffold with a reprecipitation method. We design a small scaffold material and the inoculation of suspension was 1 × 10^6^/mL, 1 to 2 mL. The cell suspension concentration is one of the critical factors that determine the inoculation rate. It is easy to form cell mass in high concentration. Meanwhile, cell number is too little to inoculate into the scaffold in low concentration. The results have been proved to be more ideal by biopsy and scanning electron microscope. Different concentrations of BAM leaching solution were added into culture medium at 1st, 2nd, 3rd, 4th, and 5th day. The RGR was greater than 80% and cell toxicity grade was 0 or 1 (Table 1), which shows that BAM has well biocompatibility with low toxicity to HFSCs and it can be used as biological scaffolds materials to re-establish tissue engineering bladder. Cell growth curve indicates that the best time to implant HFSCs–BAM complexes into animals is 7th to 8th days (Fig. 4). Scanning electron microscopy, HE staining, and masson staining show that cells have germinated on the surface of scaffold (Fig. 5). Appropriate electrical stimulation and mechanical stimulation in dynamic culture were beneficial to form natural shape and also promote mechanical characteristics of bladder to store and uresis.^[24–27]^

Regenerative medicine creates new bladder through selective cell transplantation. The success of bladder reconstruction depends on the efficiency of donor tissue and the conditions of long-term survival and differentiation. Our study demonstrates that HFSCs–BAM complexes have good biocompatibility. This discovery provide experimental basis for inducing the differentiation of HFSCs into smooth muscle and urinary epithelial cell. Further, whether exogenous inducing factors are necessary to promote tissue regeneration still need further researches. The mechanism that BAM induces the HFSCs’ directional differentiation is also the study focus in the future.
